# Supplementary material for: Exploring the Role of Osteosarcoma-Derived Extracellular Vesicles in Pre-Metastatic Niche Formation and Metastasis in the 143-B Xenograft Mouse Osteosarcoma Model
Source: Cancers (Basel). 2020 Nov 20;12(11):3457. doi: 10.3390/cancers12113457 (PMC7699714; doi:10.3390/cancers12113457)
Supplement: Supplementary file 1 [file cancers-12-03457-s001.zip › Supplementary Figures Mazumdar et al Cancers.docx]

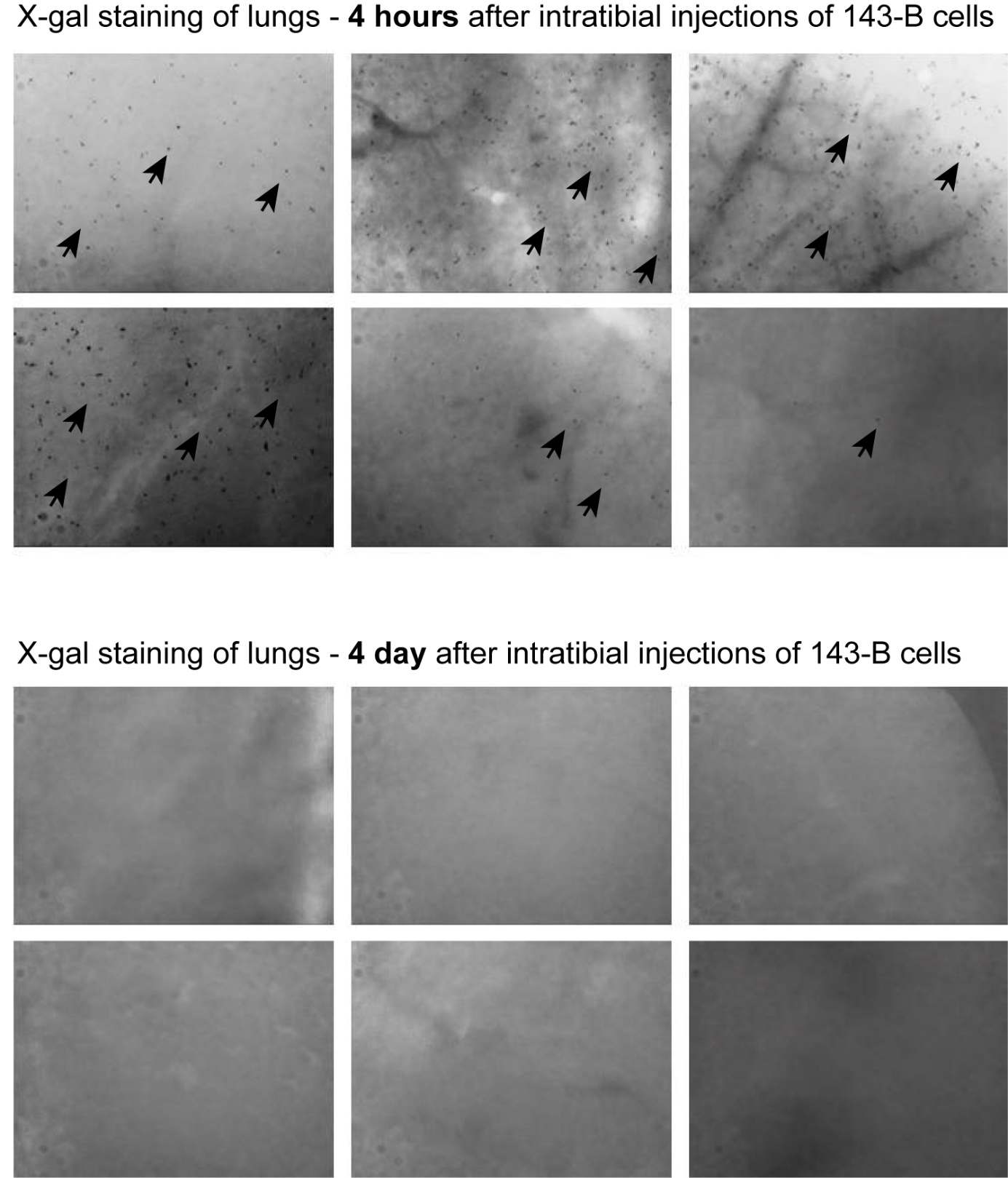


**Figure S1.** X-Gal staining of mouse lungs 4 hours and 4 days after orthotopic implantation of 143-B tumor cells. Representative pictures of individual mouse lungs (n = 6) sacrificed 4 hours (top panel) or 4 days (bottom panel, n = 6) after tumor cell injection. Black arrows indicate tumor cells. Scale bar, 500 µm.


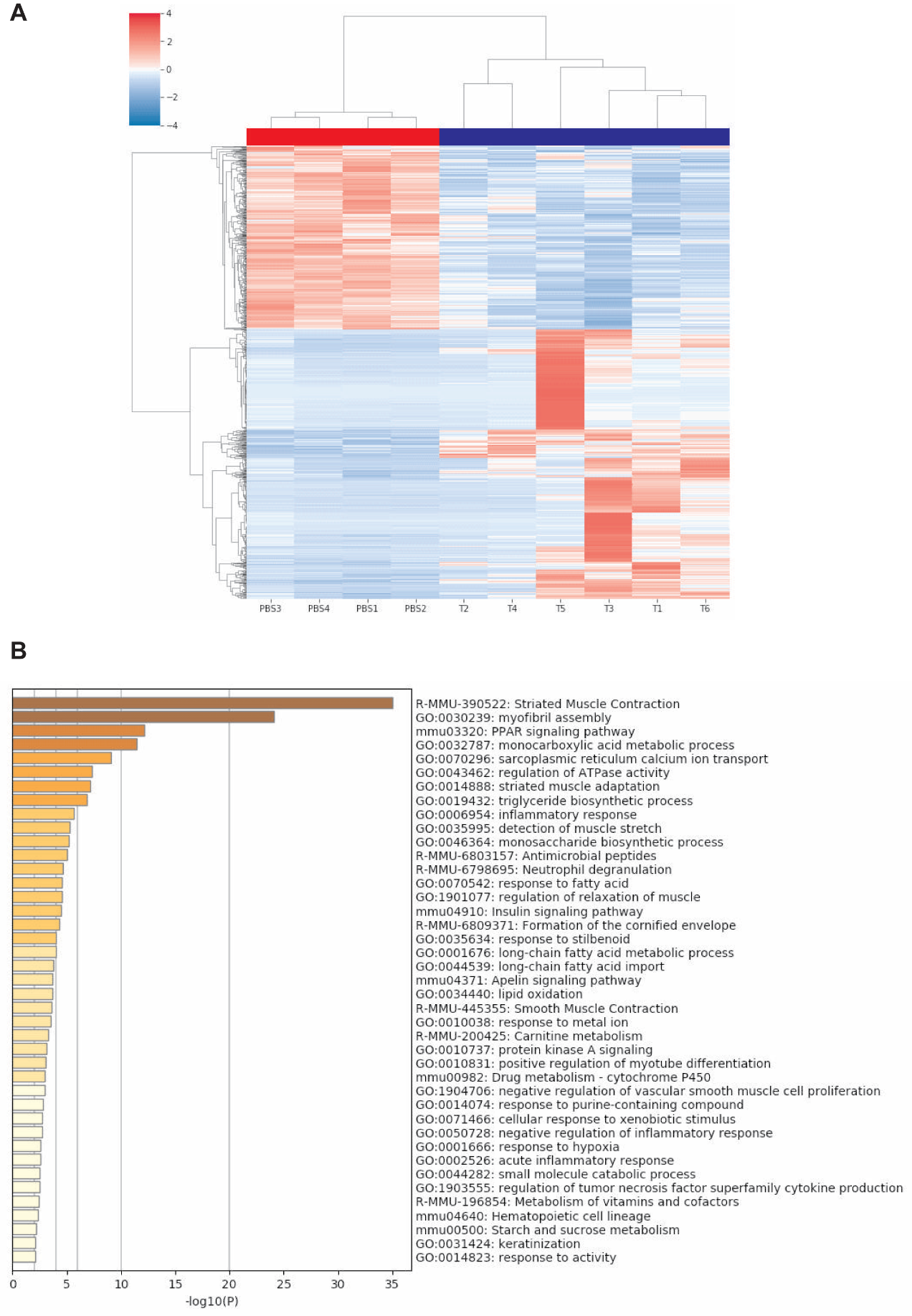
**Figure S2.** Transcriptomic profiling of pre-metastatic lungs. (**a**) Unsupervised hierarchical clustering of all genes. Each column represents one biological replicate (n = 6 in the tumor group(T1-T6), n = 4 in the control group (PBS1-PBS4)). Cut-off values of fold change are >1.5 and FDR <0.2. The clustering separates the genes by color with positive or negative row-scaled Z-scores represented in red and blue, respectively. (**b**) Metascape analysis of the top 250 upregulated genes.

**
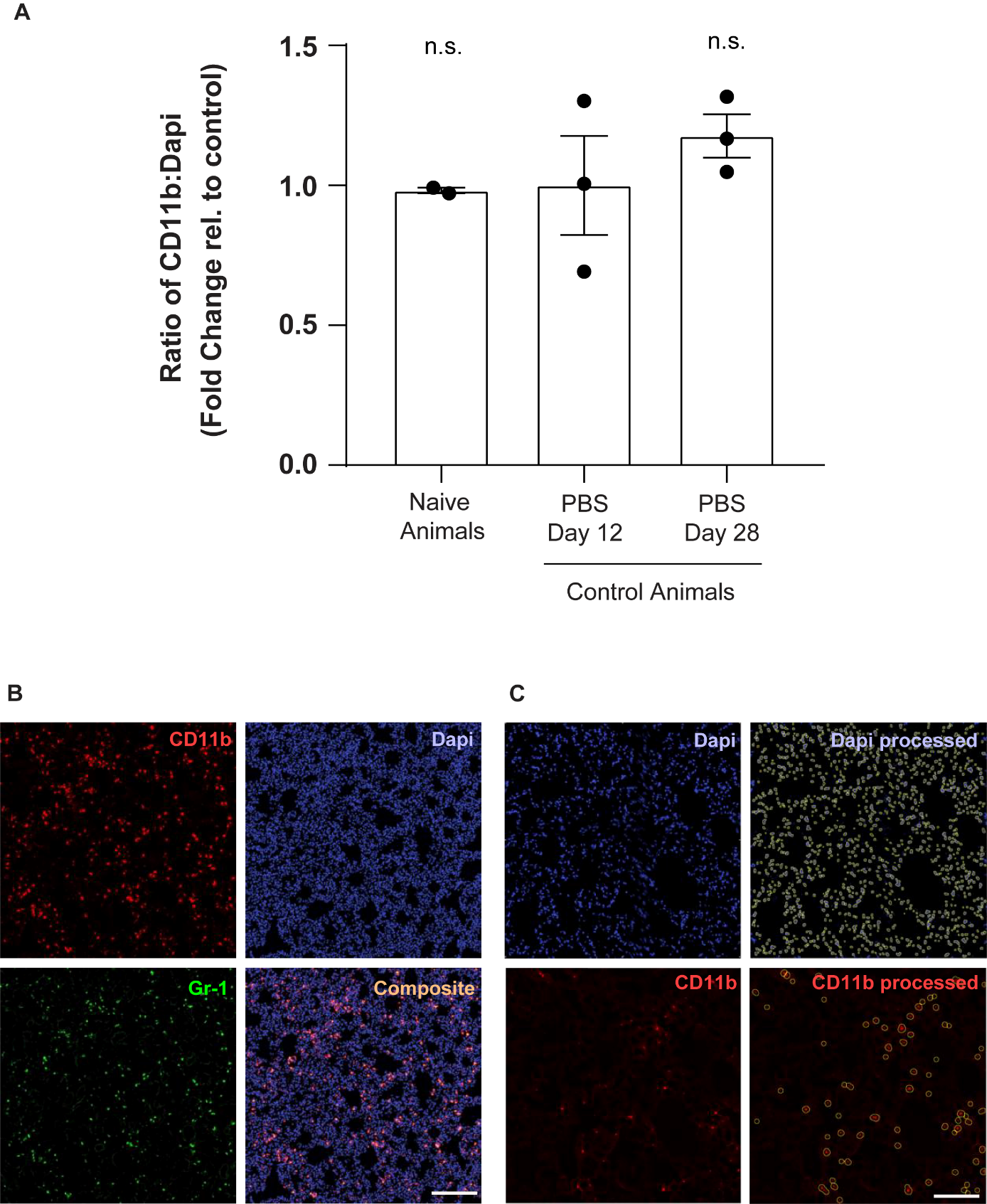
Figure S3.** CD11b^+^ and Gr-1 expression in mouse lung sections. (**a**) Quantification of CD11b^+^ cells in the indicated mouse lung sections from the experiment presented in Figure 2. Groups of control mice were intratibially injected with PBS on day 0 and sacrificed on the above indicated dates. The data is normalized to PBS day 12. (n.s., non-significant; Bonferroni's one-way ANOVA test) (**b**) Representative images of CD11b^+^ (red), Gr-1 (green), Dapi (blue) and the composite of mice lungs educated with 143-B EVs for 3 weeks as presented in Figure 3. (**c**) Quantification of CD11b^+^ cells (red) and nuclei (blue) in the representative images shown in (b). Quantification of nuclei was used to normalize the number of CD11b+ positive cells. Scale bar, 250 µm.

**
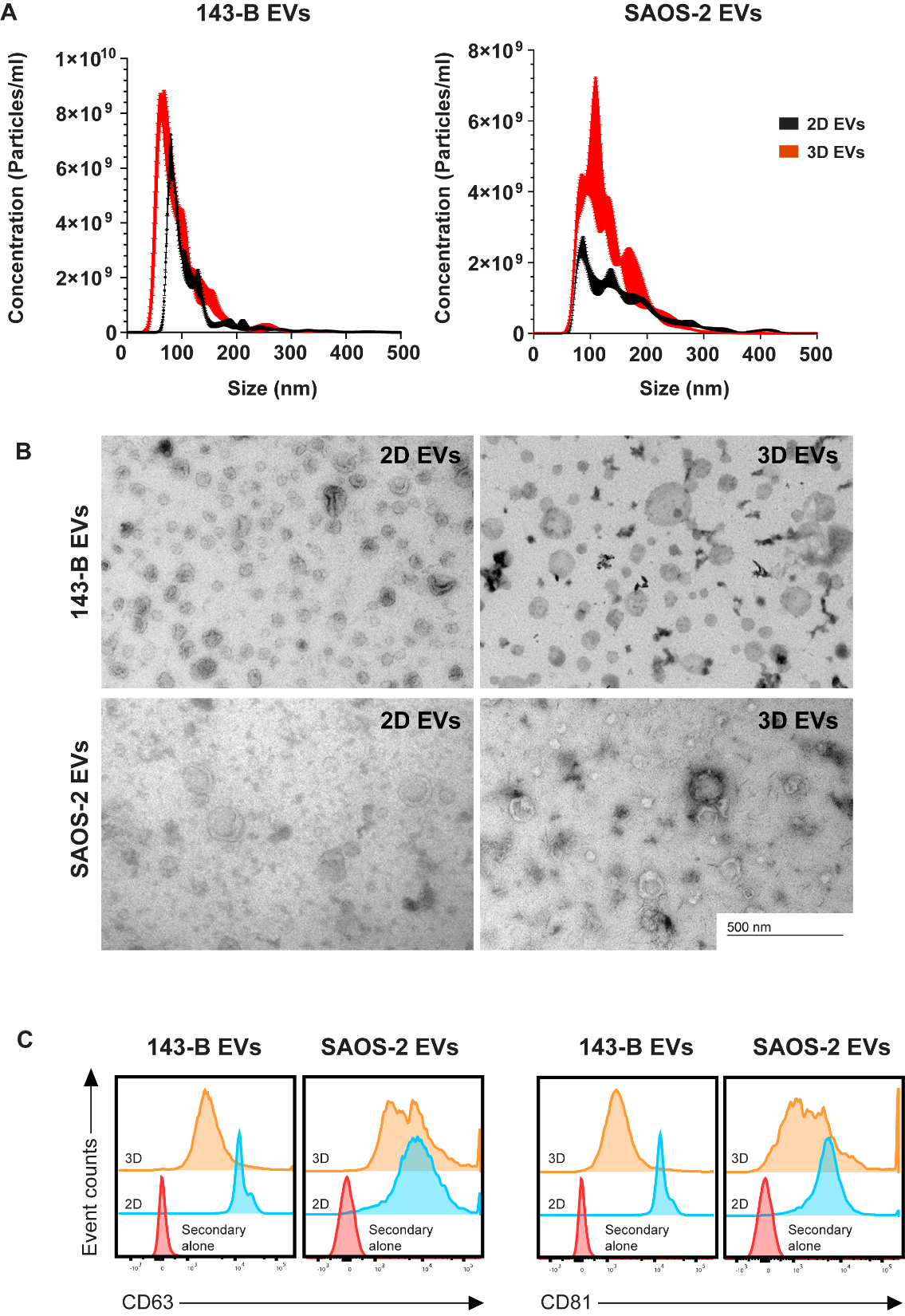
**

**Figure S4.** Characterization of osteosarcoma 143-B- and SaOS-2 derived extracellular vesicles (EVs) (**a**) EV size distribution assessed by NanoSight analysis; (**b**) Transmission electron micrographs of 143-B and SaOS-2 EVs. (**c**) Representative histograms of flow cytometry analysis of exosomal markers CD63 and CD81 in 143-B and SaOS-2 EV-coated beads.
